# Supplementary material for: Predicting higher-order mutational effects in an RNA enzyme by machine learning of high-throughput experimental data
Source: Front Mol Biosci. 2022 Aug 15;9:893864. doi: 10.3389/fmolb.2022.893864 (PMC9421044; doi:10.3389/fmolb.2022.893864)
Supplement: Supplementary file 1 [file DataSheet1.PDF]

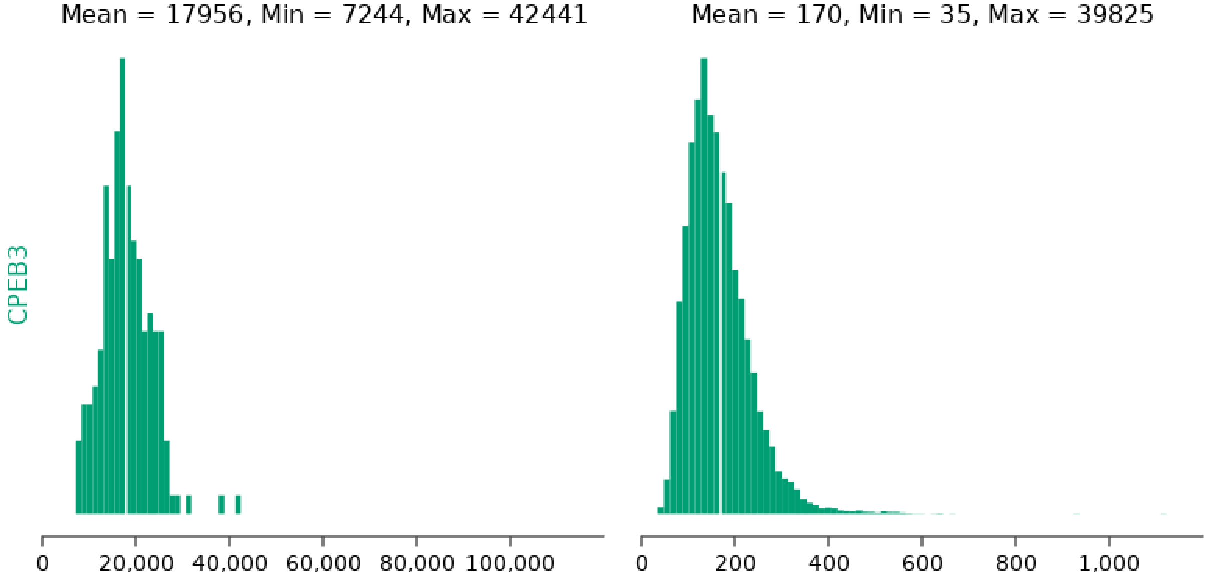

Supplementary Figure 1. Histogram of CPEB3 variant counts for single (left) and double (right) mutants. Mean, minimum and maximum values for each distribution are indicated.

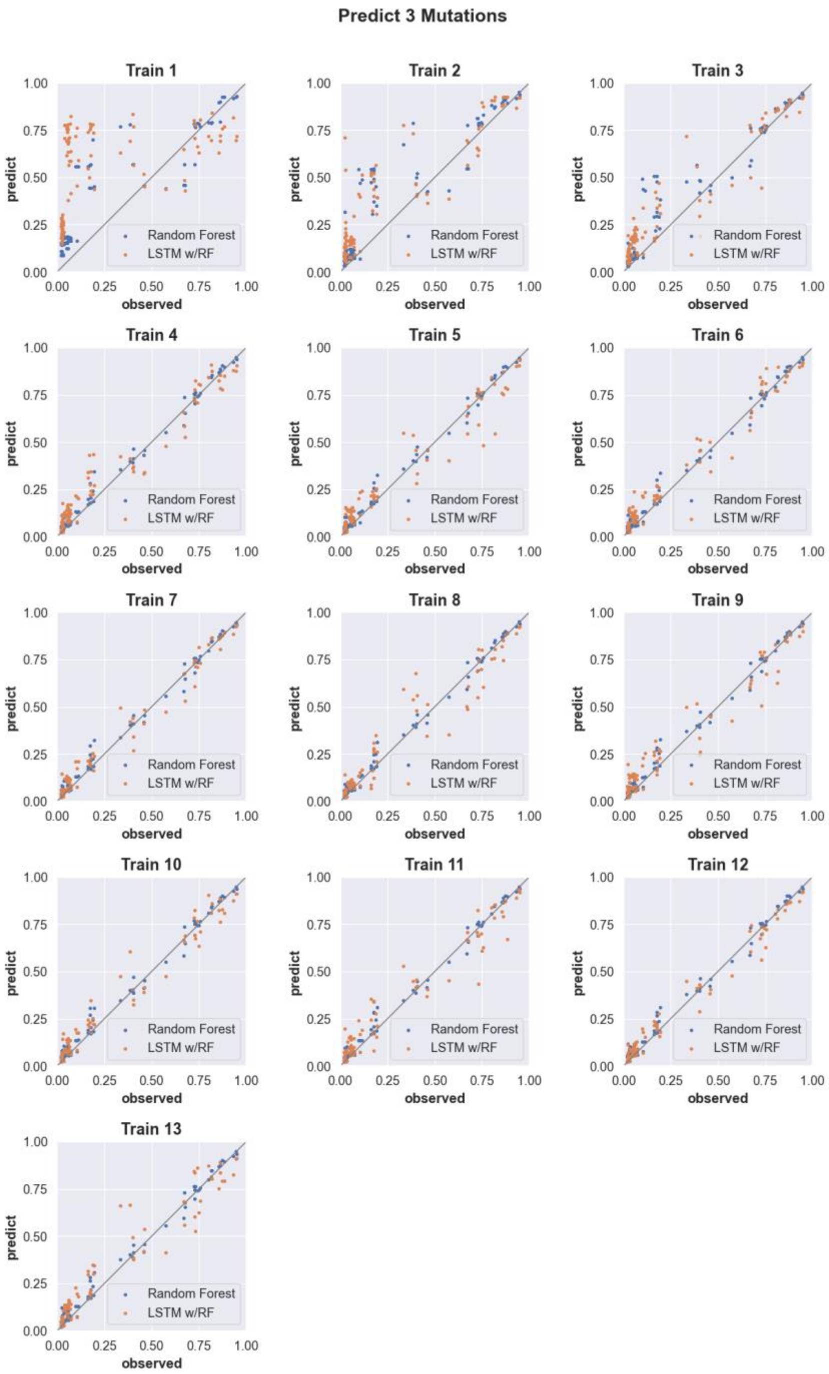

Supplementary Figure 2. Three-mutation sequence activity predictions. Scatter plots comparing fraction cleaved values measured from experiments (observed) to those predicted by models (predict) trained by either random forest (blue) or the LSTM approach (orange). Each scatter plot shows the predictions from a different training data set. The training data contained sequences with up to the number of mutations in the title (Train N). For example, ‘Train 5’ indicates that the model was trained using data for sequences containing 1,2,3,4, and 5 mutations. The line indicates unity, not a fit to the data.

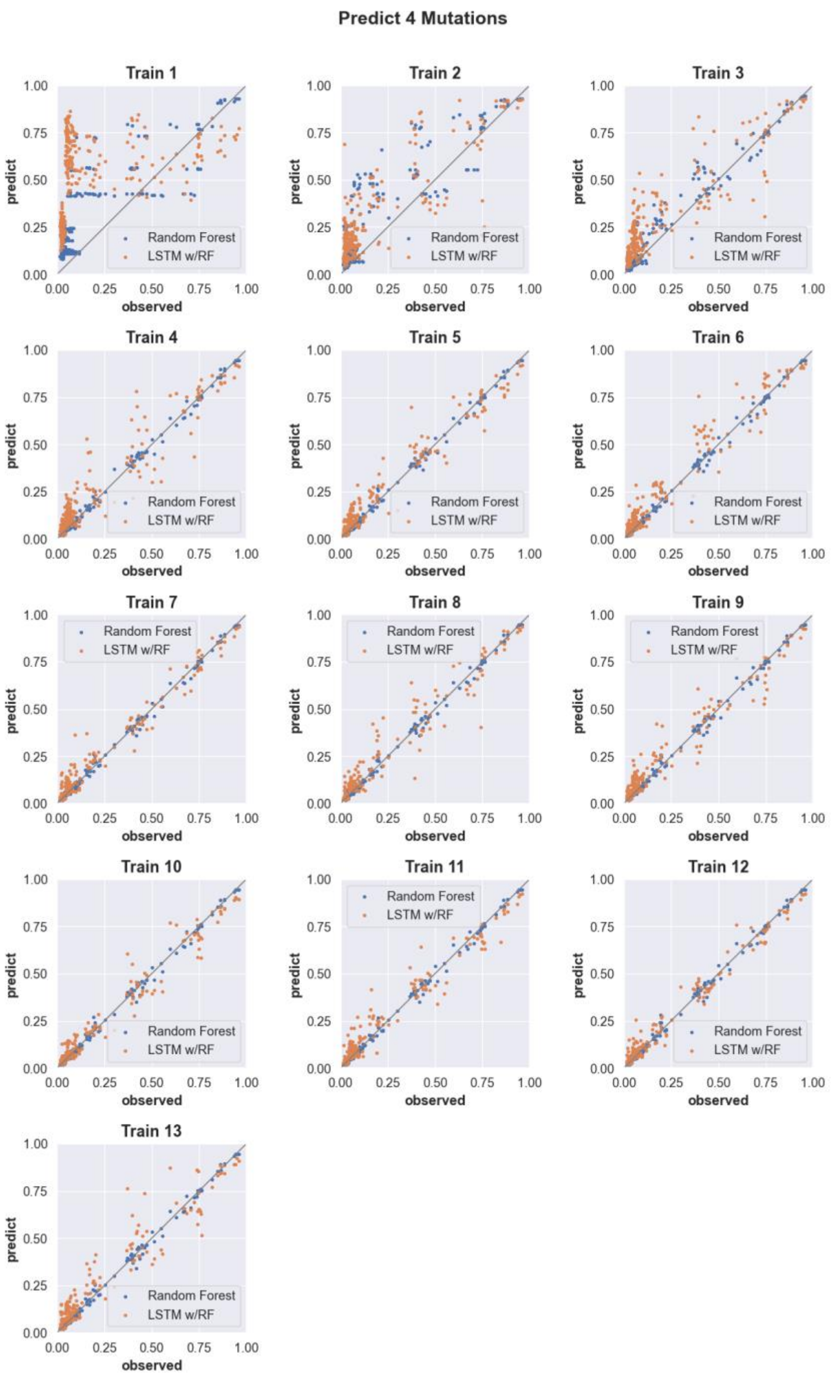

Supplementary Figure 3. Predicting the activity of sequences with four mutations (see Supp. Fig. 2 for details).

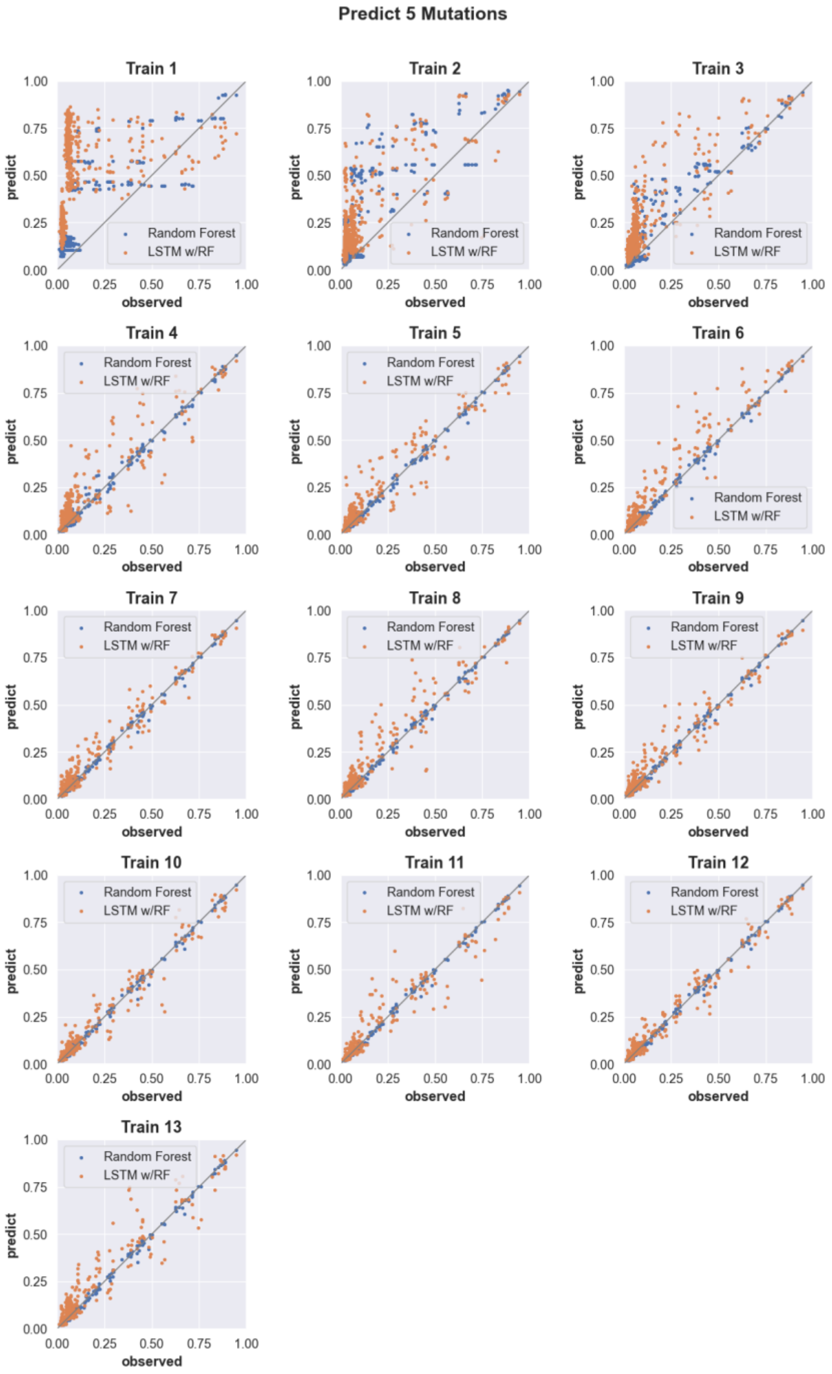

Supplementary Figure 4. Predicting the activity of sequences with five mutations. (see Supp. Fig. 2 for details).

.

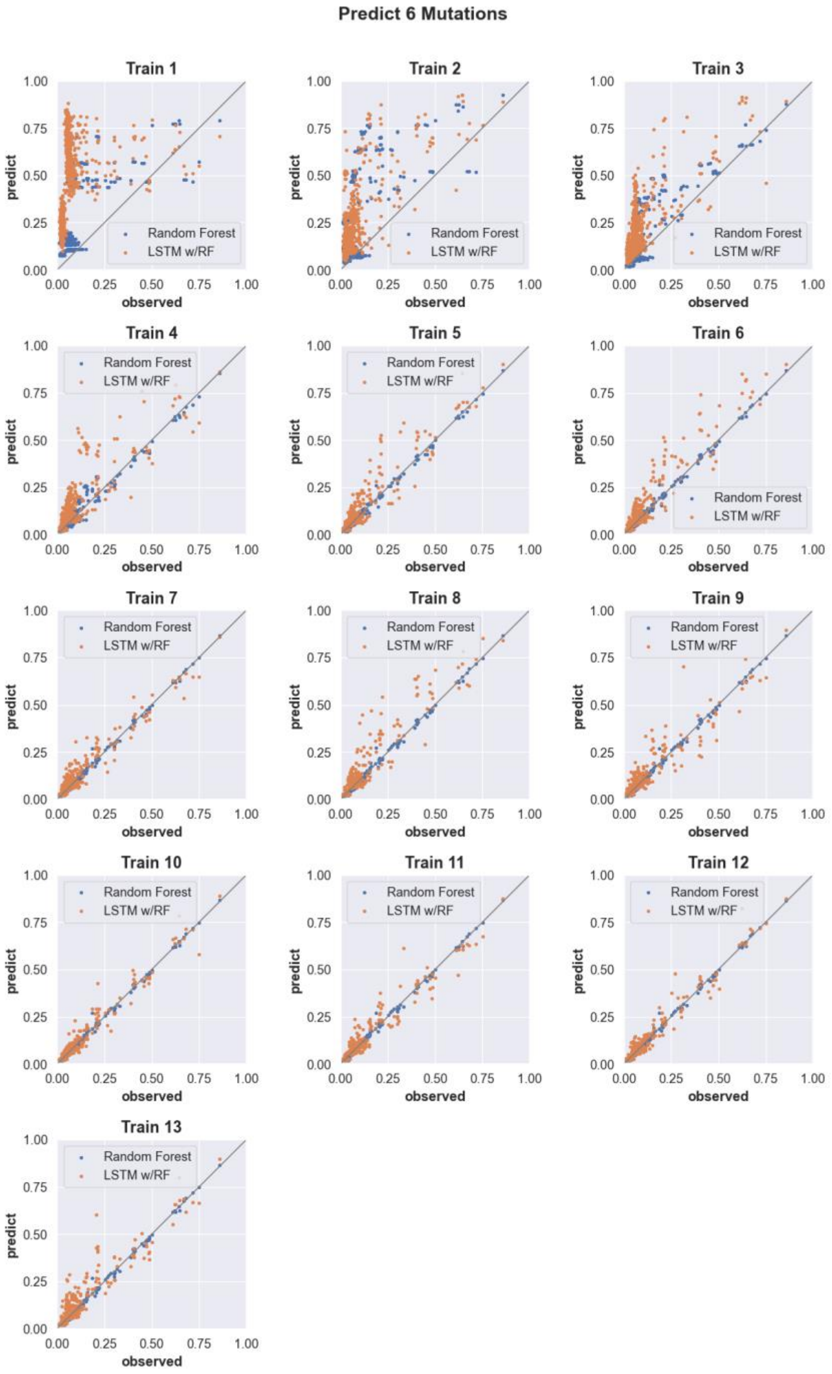

Supplementary Figure 5. Predicting the activity of sequences with six mutations (see Supp. Fig. 2 for details).

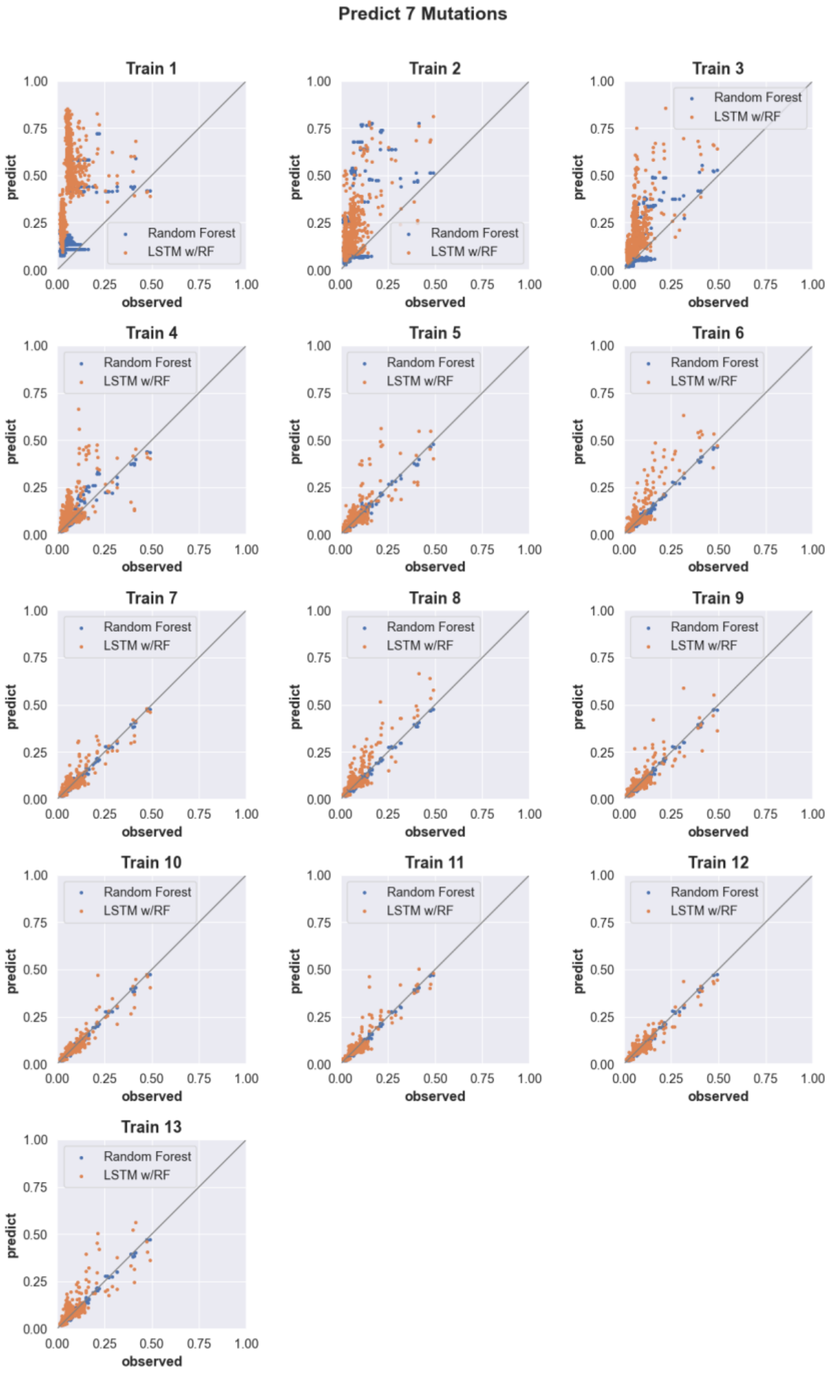

Supplementary Figure 6. Predicting the activity of sequences with seven mutations (see Supp. Fig. 2 for details).

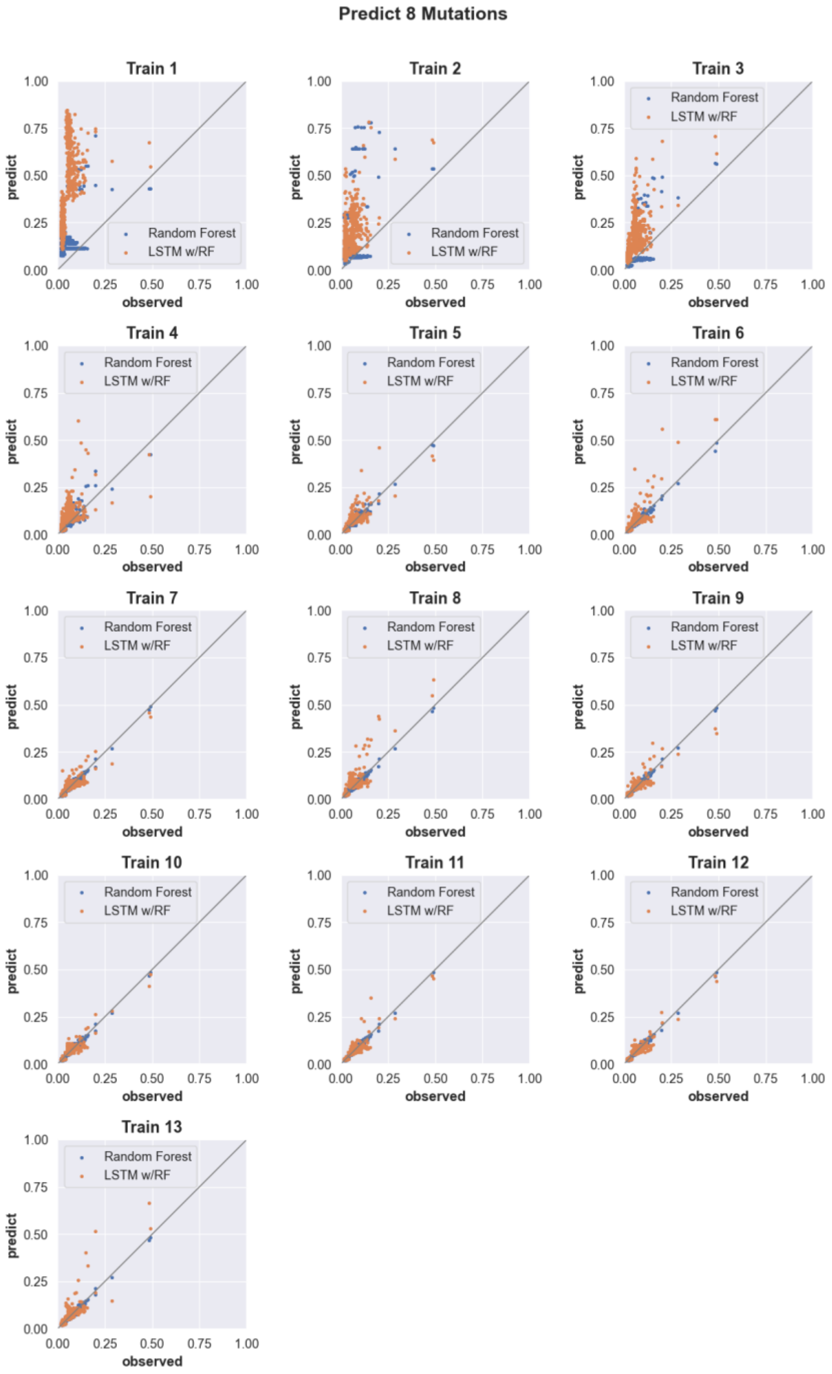

Supplementary Figure 7. Predicting the activity of sequences with eight mutations (see Supp. Fig. 2 for details).

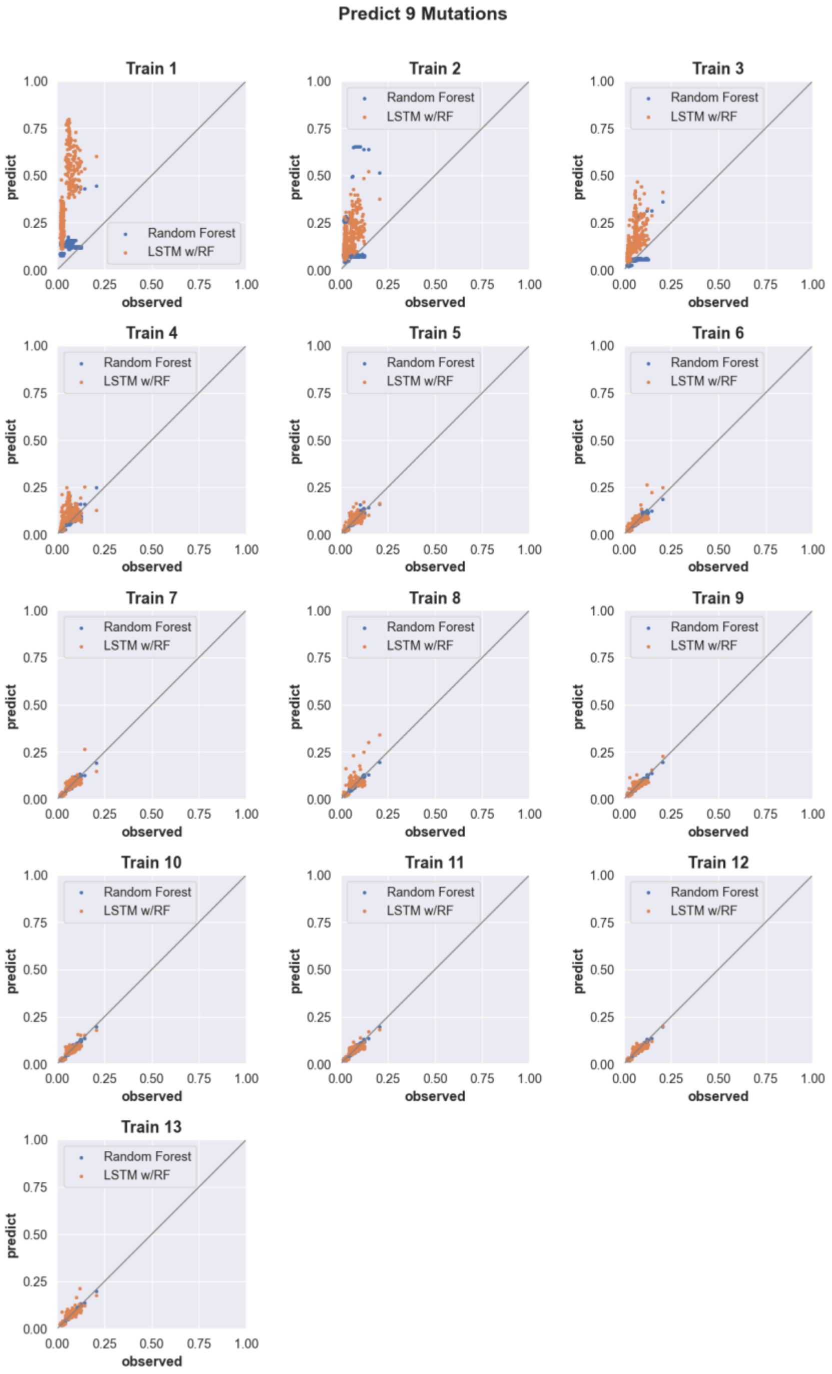

Supplementary Figure 8. Predicting the activity of sequences with nine mutations (see Supp. Fig. 2 for details).

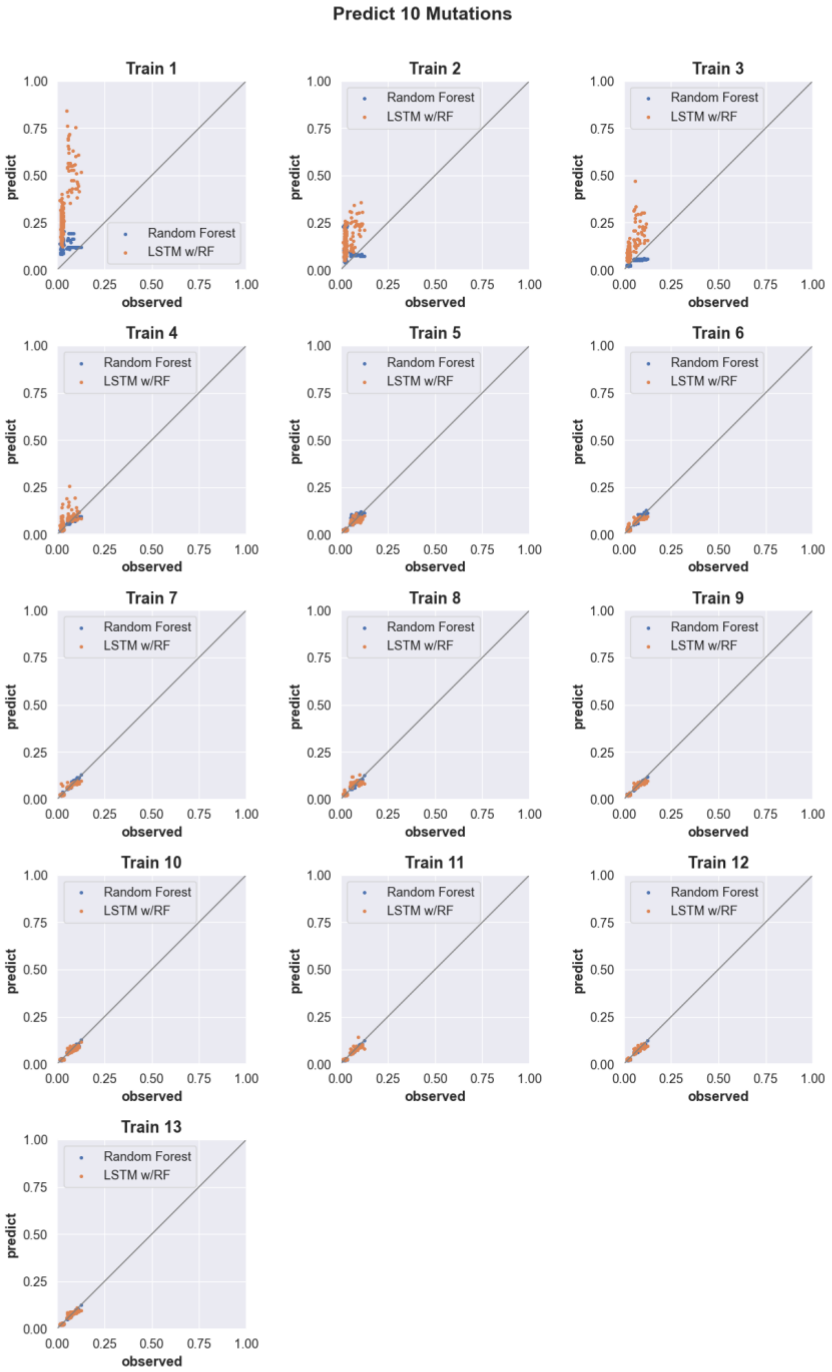

Supplementary Figure 9. Predicting the activity of sequences with 10 mutations (see Supp. Fig. 2 for details).

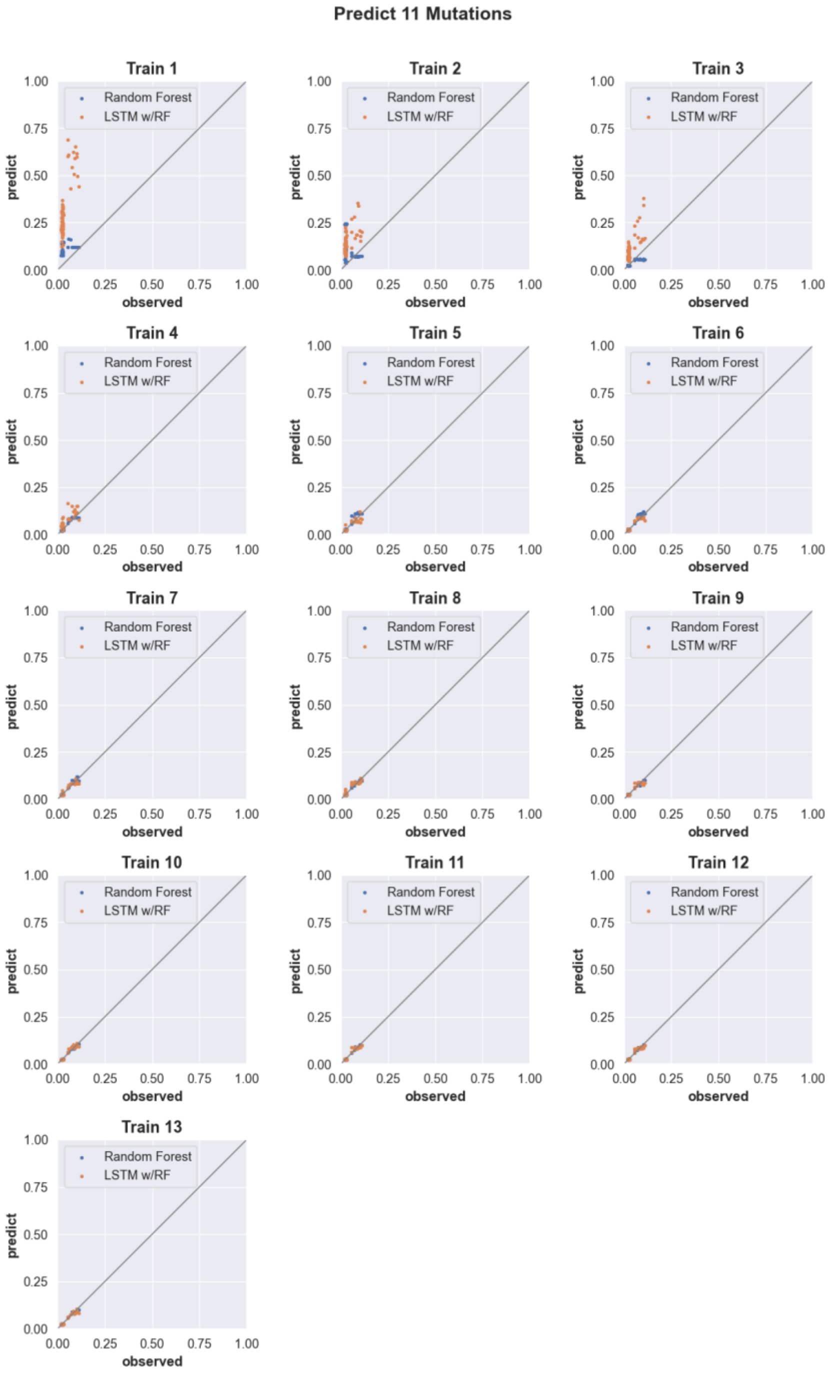

Supplementary Figure 10. Predicting the activity of sequences with 11 mutations (see Supp. Fig. 2 for details).

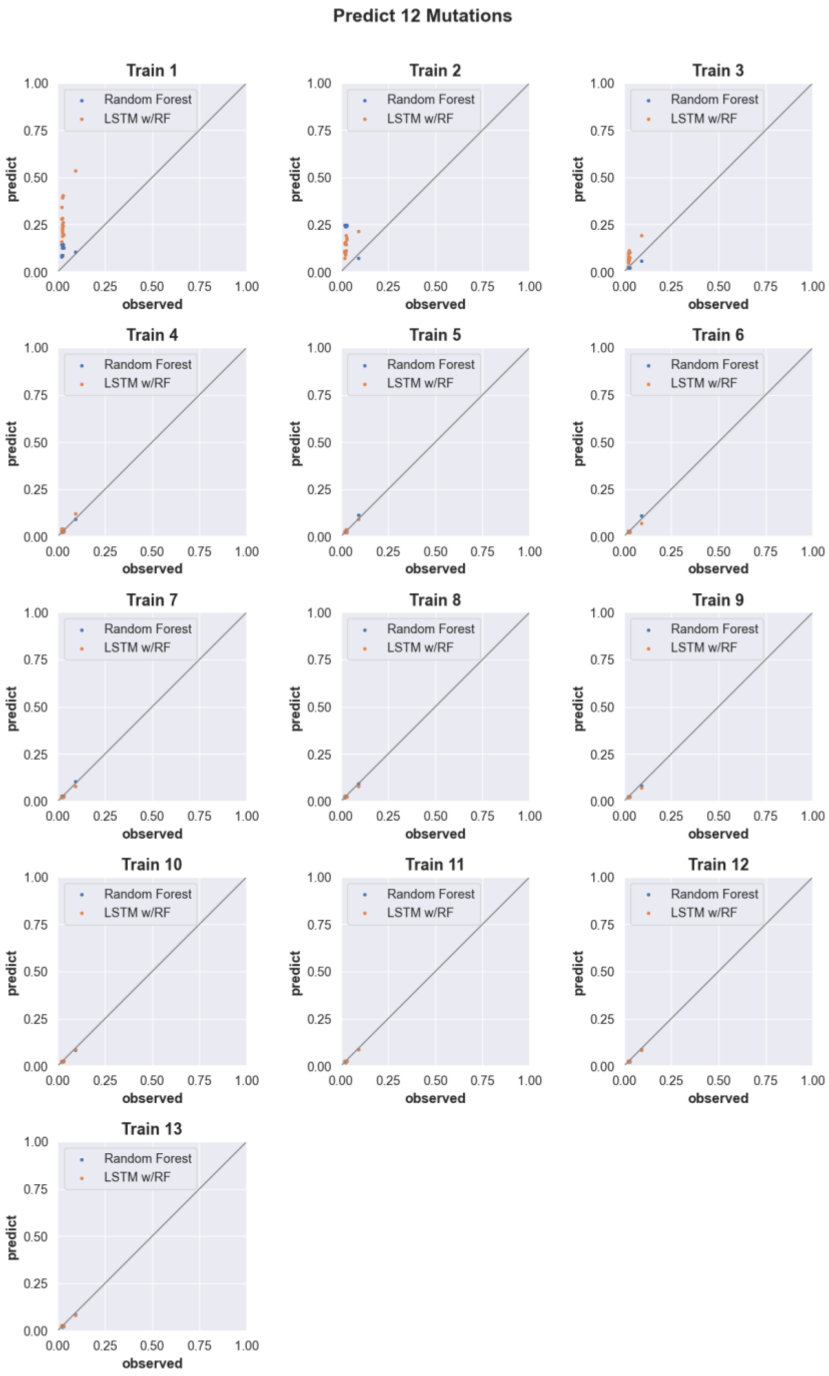

Supplementary Figure 11. Predicting the activity of sequences with 12 mutations (see Supp. Fig. 2 for details).

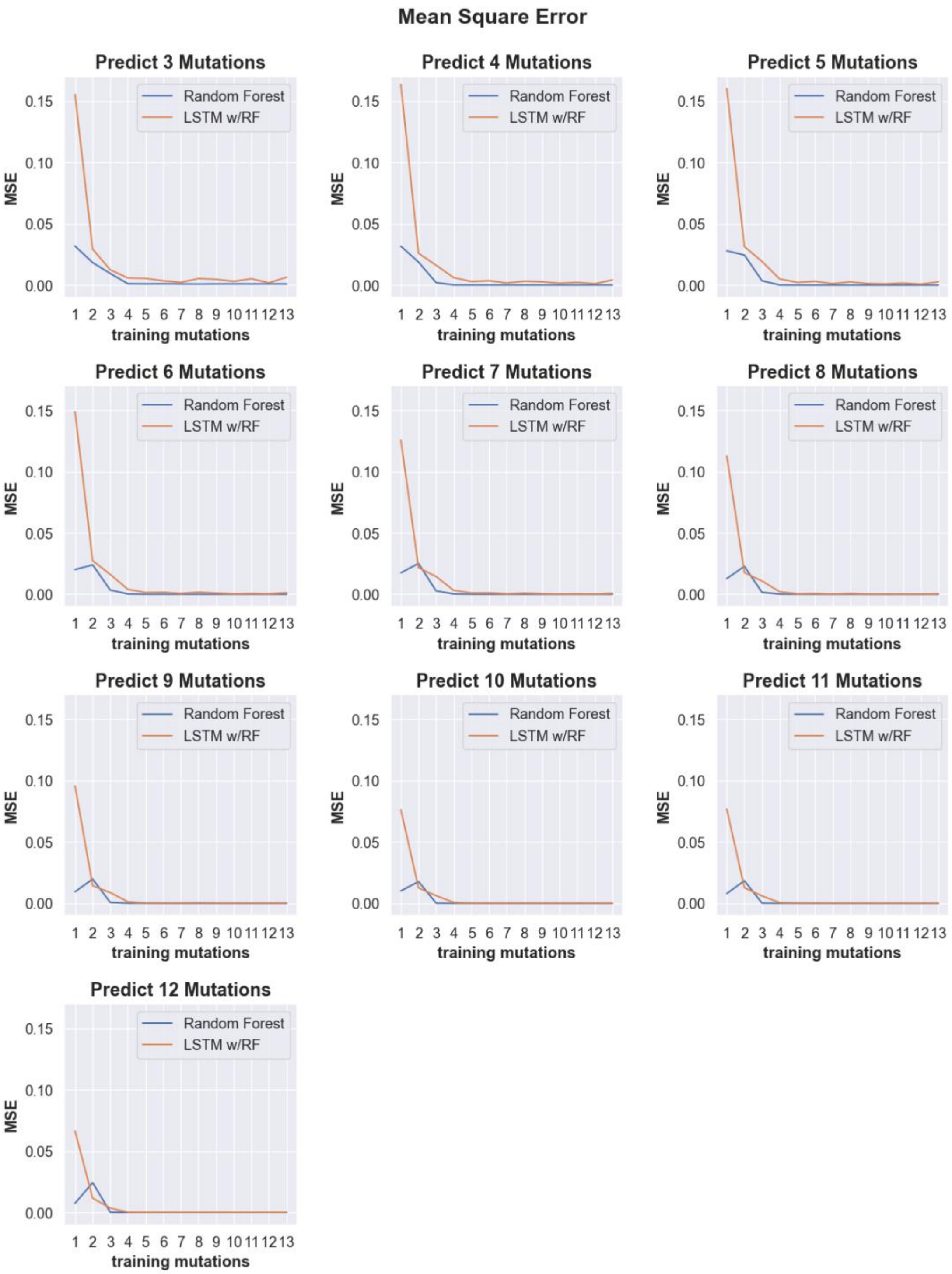

Supplementary Figure 12. Line plots showing the mean square error (MSE) of predicted cleavage activity values obtained from random forest (blue) and LSTM with random forest (orange) machine learning models trained on data with incrementally increasing numbers of mutations shown along the x-axis. Each plot shows the MSE for predictions obtained for sequences containing the number of mutations indicated by the plot title.

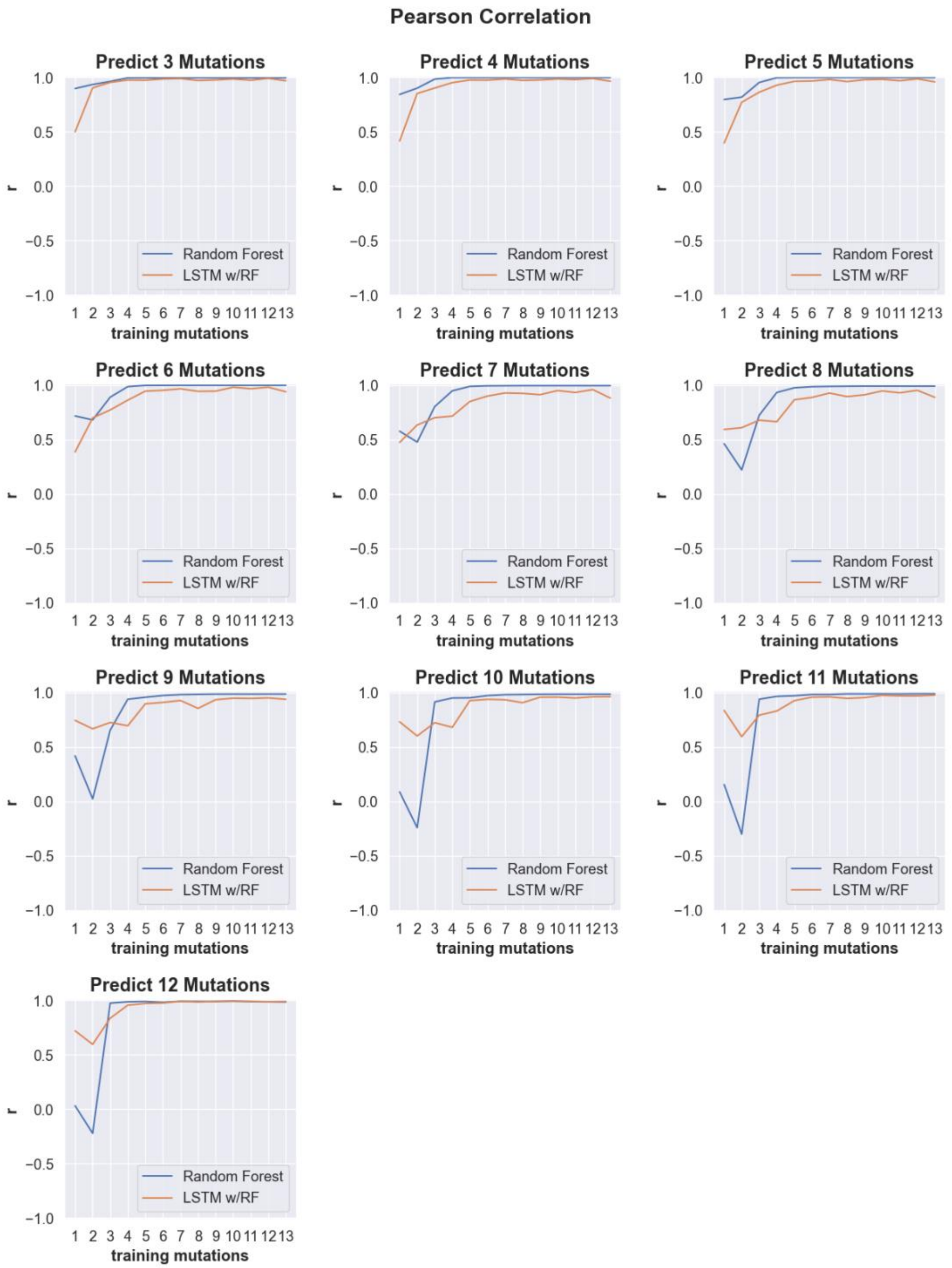

Supplementary Figure 13. Line plots showing the Pearson correlation values of predicted cleavage activity obtained from random forest (blue) and LSTM with random forest (orange) machine learning models trained on data with incrementally increasing numbers of mutations shown along the x-axis. Each plot shows the Pearson correlation for predictions obtained for sequences containing the number of mutations indicated by the plot title.

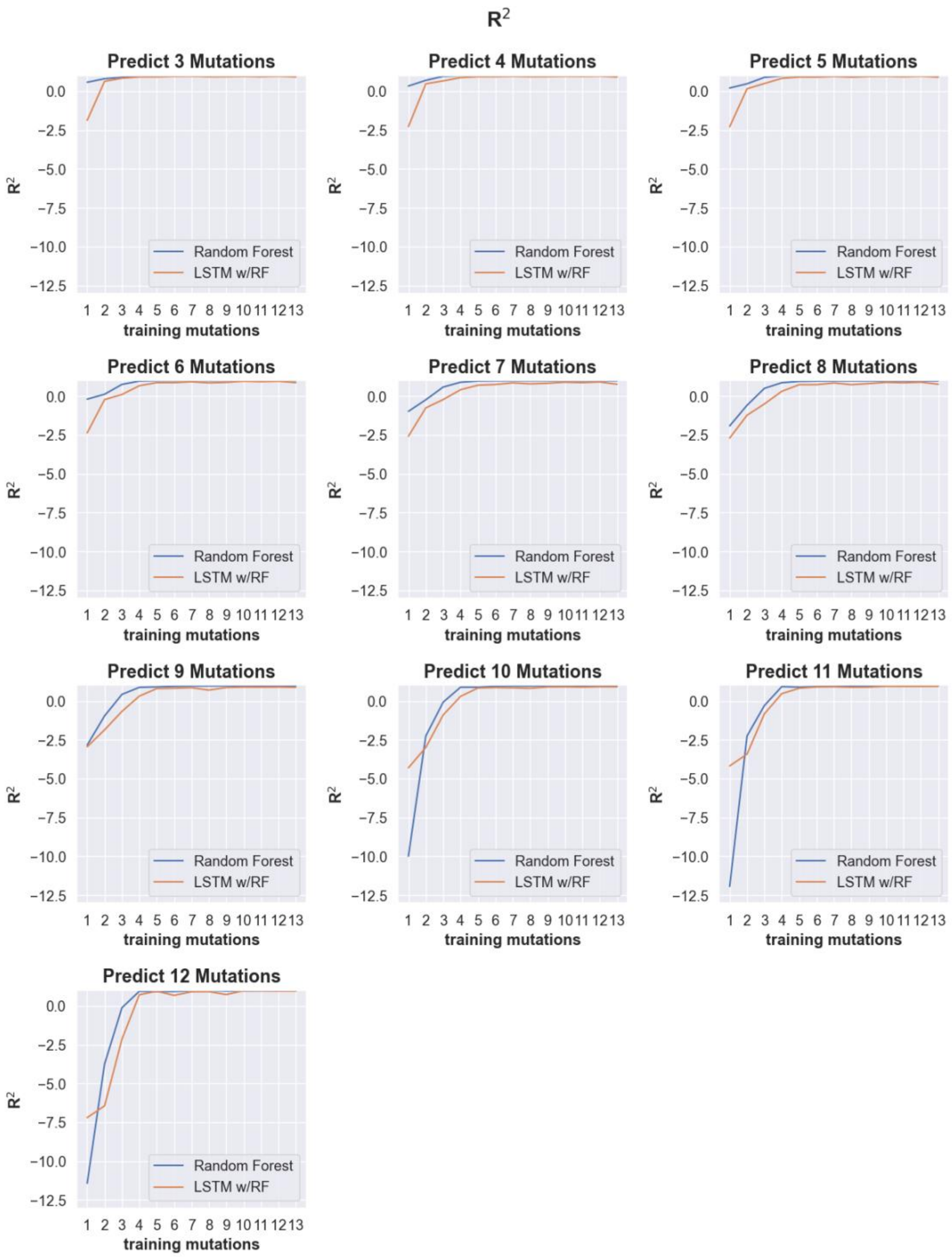

Supplementary Figure 14. Line plots showing the R^2 values of predicted cleavage activity obtained from random forest (blue) and LSTM with random forest (orange) machine learning models trained on data with incrementally increasing numbers of mutations shown along the x-axis. Each plot shows the R^2 for predictions obtained for sequences containing the number of mutations indicated by the plot title.

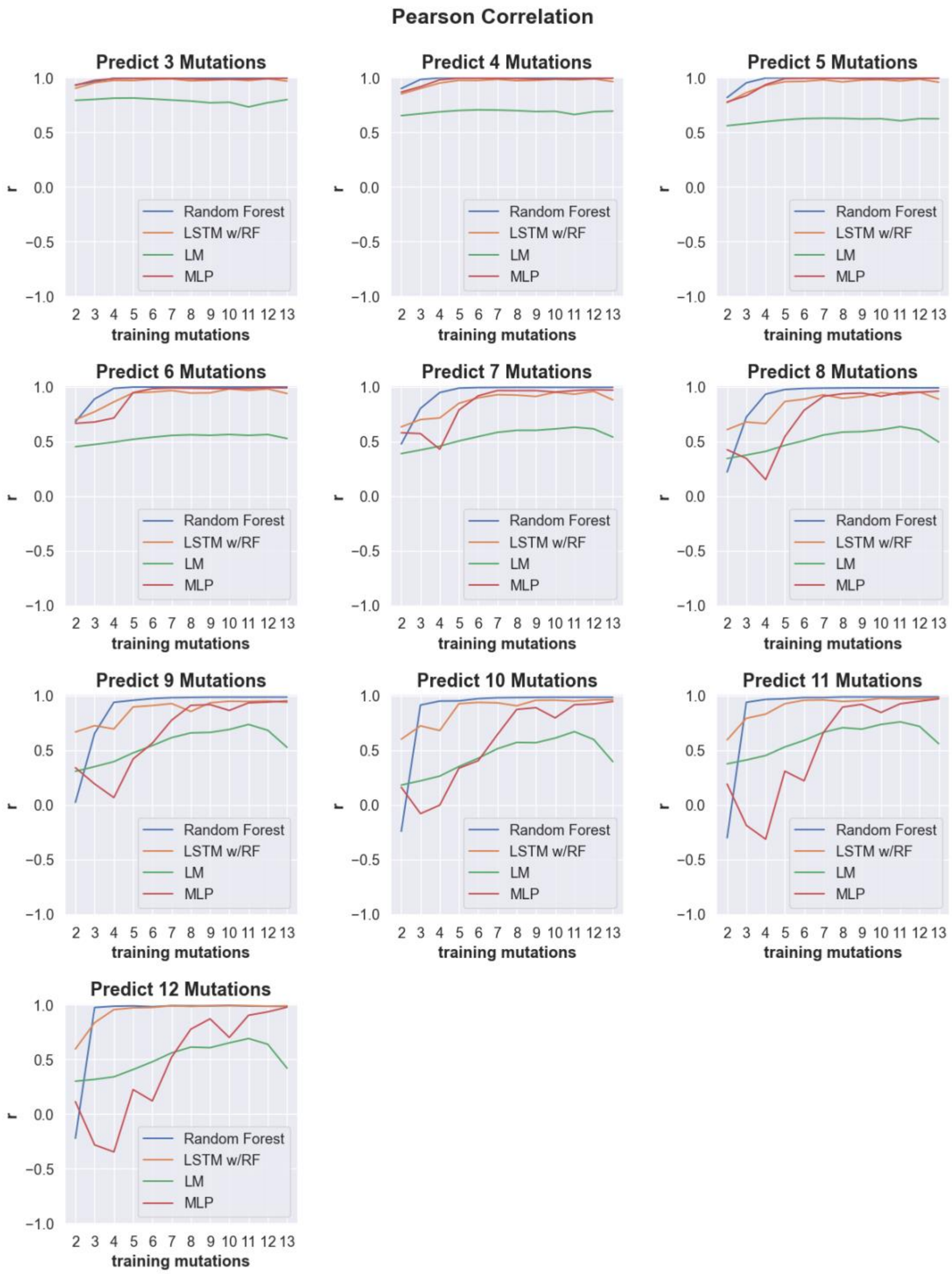

Supplementary Figure 15. Benchmarking against several ML approaches. Line plots showing the Pearson correlation values of predicted cleavage activity obtained from random forest (blue), LSTM with random forest (orange), linear regression (green) and multilayer perceptron regressor (red) machine learning models trained on data with incrementally increasing numbers of mutations shown along the x-axis. Each plot shows the Pearson correlation for predictions obtained for sequences containing the number of mutations indicated by the plot title.

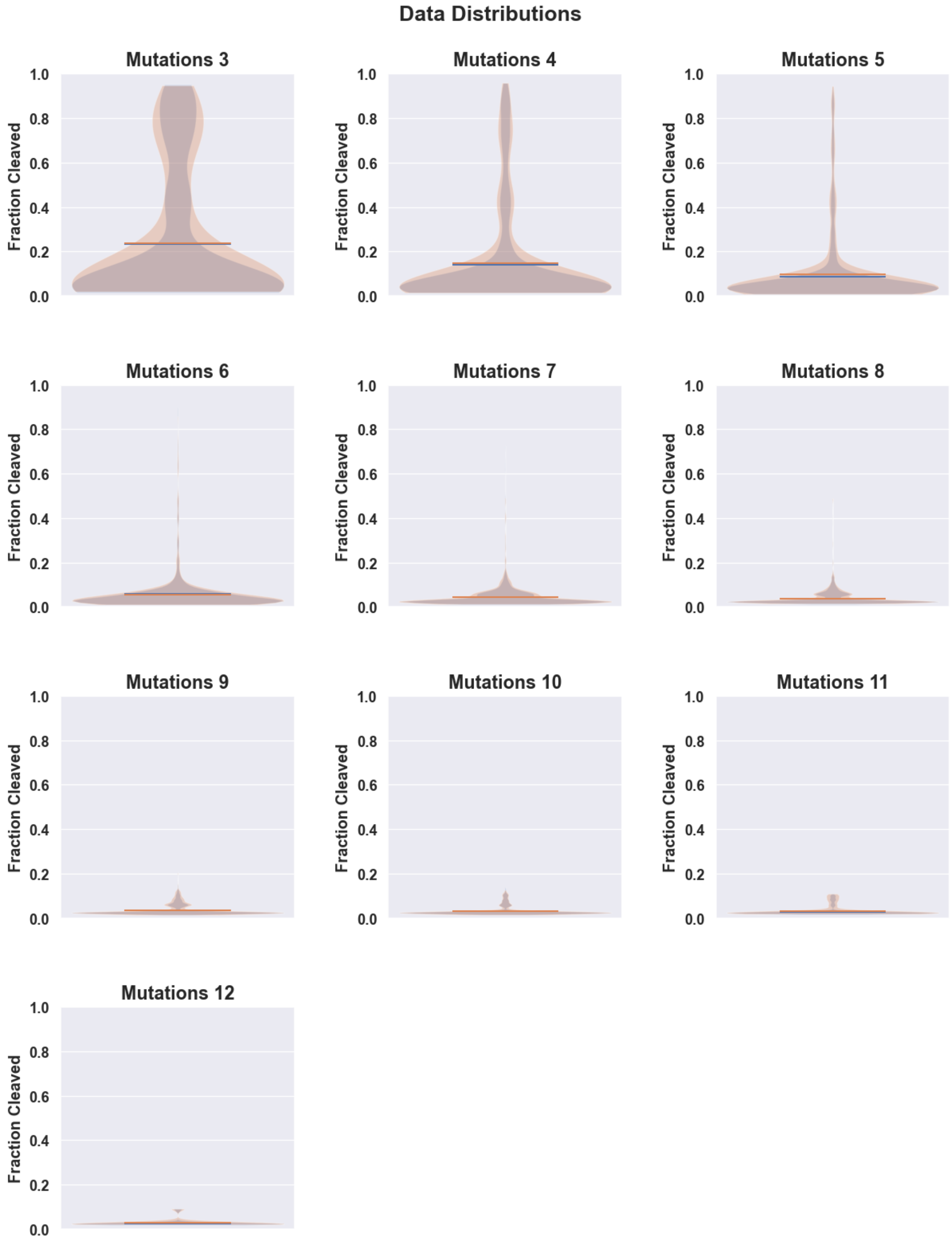

Supplementary Figure 16. Violin plots showing the distribution of cleavage rates observed in the test data (orange) and the total data set for a given mutation (blue). The

distributions are shown separately for each data set containing increasing numbers of mutations, from 3 to 12.

A

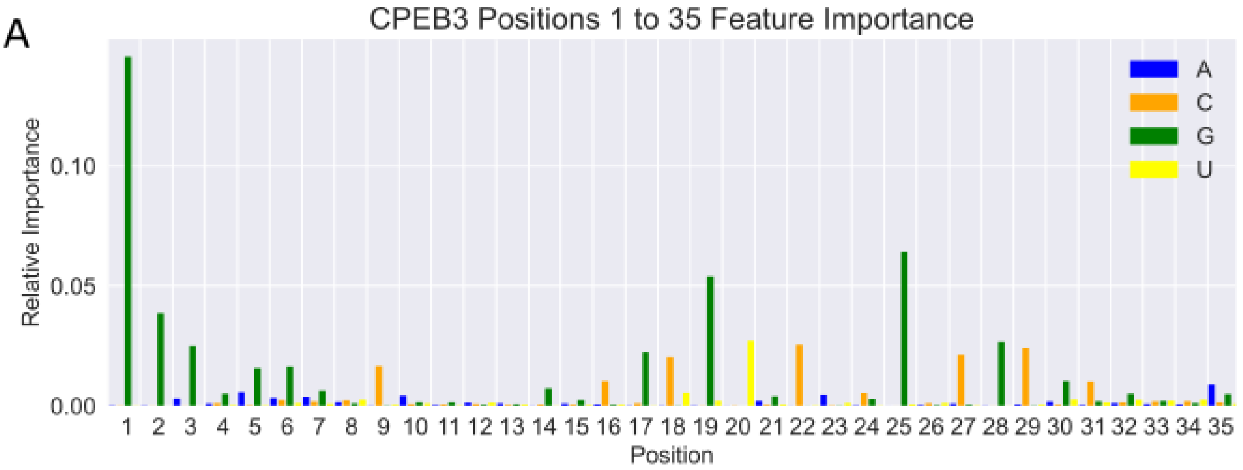

B

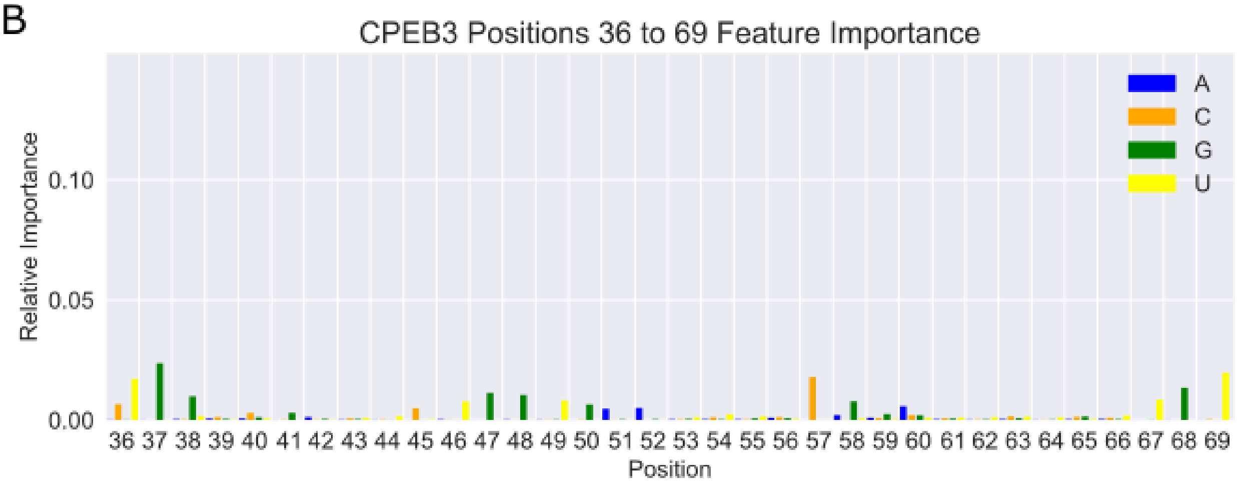

C

Top Ten Feature Importance

Rank	Train 2	Train 3	Train 4	Train 5	Train 6	Train 7	Train 8	Train 9	Train 10	Train 11	Train 12	Train 13
1	G2:0.044	G1:0.049	G1:0.084	G1:0.145	G1:0.207	G1:0.251	G1:0.278	G1:0.294	G1:0.301	G1:0.304	G1:0.305	G1:0.305
2	G1:0.041	G2:0.046	G25:0.046	G25:0.064	G25:0.082	G25:0.093	G25:0.096	G25:0.1	G19:0.096	G25:0.101	G25:0.098	G25:0.096
3	G25:0.034	G25:0.037	G2:0.043	G19:0.054	G19:0.068	G19:0.08	G19:0.089	G19:0.089	G25:0.095	G19:0.09	G19:0.092	G19:0.095
4	T20:0.034	G28:0.034	G19:0.042	G2:0.039	G2:0.032	G2:0.028	G2:0.027	G2:0.026	G2:0.026	G2:0.026	G2:0.026	G2:0.026
5	G28:0.033	T20:0.034	T20:0.032	T20:0.027	C29:0.024	C29:0.025	C29:0.024	C29:0.024	C29:0.023	C29:0.021	C29:0.022	C29:0.02
6	C22:0.033	G19:0.033	G28:0.032	G28:0.027	T20:0.024	T20:0.021	T20:0.019	T20:0.019	T20:0.018	T20:0.018	T20:0.018	T20:0.018
7	G19:0.032	C22:0.033	C22:0.03	C22:0.026	G28:0.024	G28:0.021	G28:0.019	G28:0.018	G28:0.018	G28:0.018	G28:0.018	G28:0.018
8	G3:0.032	G3:0.032	G3:0.029	G3:0.025	C22:0.023	C22:0.02	C22:0.018	C22:0.018	C22:0.017	C22:0.017	C22:0.017	C22:0.017
9	G37:0.03	G37:0.029	G37:0.027	C29:0.024	G3:0.022	G3:0.019	G3:0.018	G3:0.017	G3:0.017	G3:0.017	G3:0.017	G3:0.017
10	T69:0.029	T69:0.026	G17:0.024	G37:0.024	G37:0.02	G37:0.018	T36:0.017	T36:0.016	T36:0.016	T36:0.016	T36:0.016	T36:0.015
Total	0.342	0.353	0.387	0.454	0.527	0.576	0.606	0.621	0.628	0.627	0.629	0.627

Supplementary Figure 17. Summary of important features extracted from random forest models. A-B) Bar graphs of feature importance when training with up to five mutations. Each feature represents a specific nucleotide at a specific location, as indicated by the X-axis label (position), and color (nucleotide identity). Positions 1-35 are shown in (A), and positions 36-69 are shown in (B). The height of the bar indicates the relative importance. C) Table ranking the top ten important features extracted from random forest models trained with increasing numbers of mutations. Nucleotides discussed in the main text are highlighted.

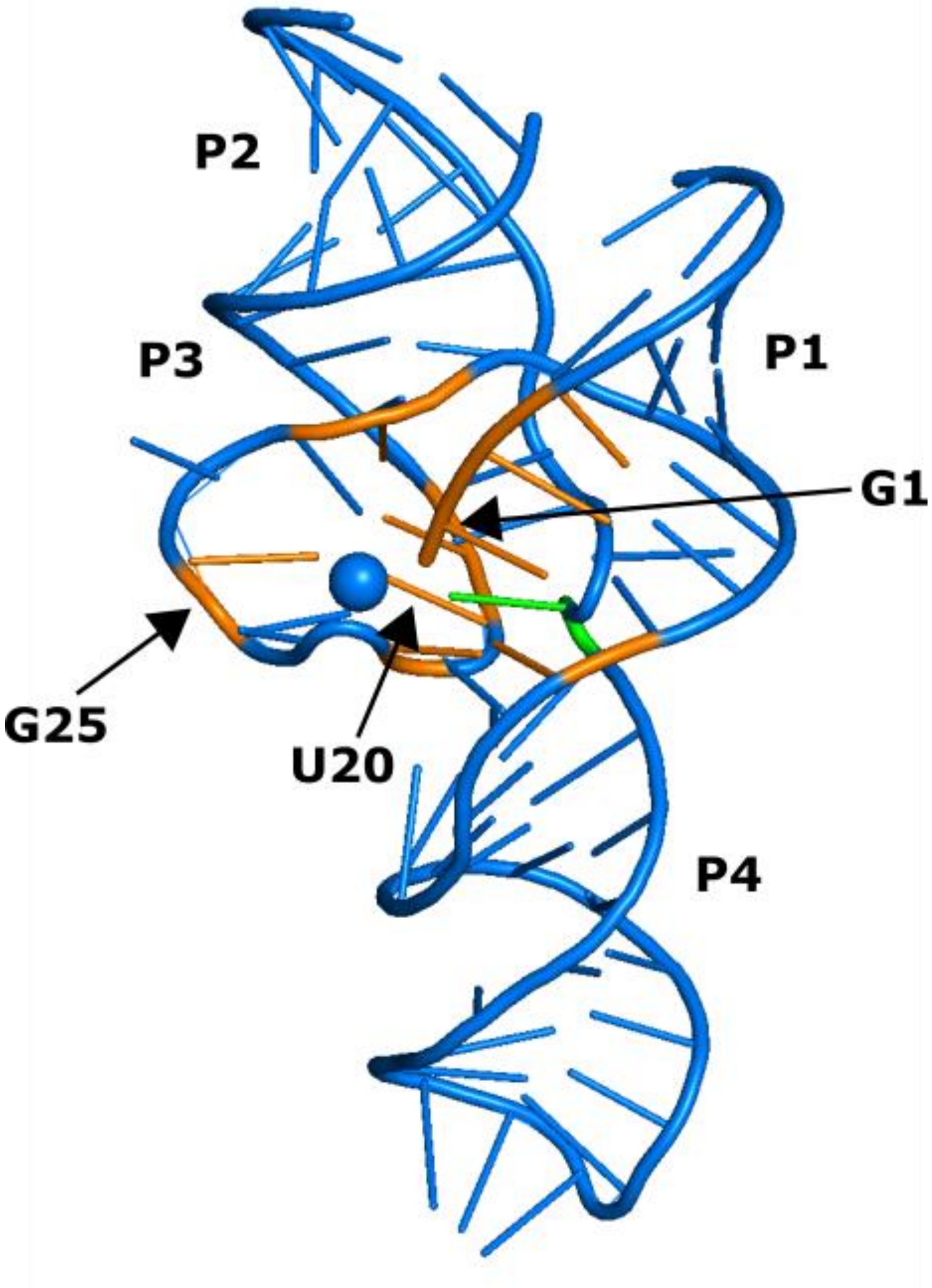

Supplementary Figure 18. Crystal structure of an HDV ribozyme (PDB 3NKB) showing the CPEB3 analogous positions representing the top ten important features identified in our random forest models. The feature importance depicted was extracted from the random forest model trained on CPEB3 data including up to 5 mutations. The nucleotides identified as the top ten important features are shaded in orange, the catalytic nucleotide is shaded green (C57/75), and the catalytic Mg^{2+} ion is depicted as a blue sphere.

Supplemental Material

Train with %	Pearson	Spearman
80%	0.99	0.81
60%	0.99	0.80
40%	0.97	0.79
20%	0.89	0.80
10%	0.91	0.77
1%	0.81	0.71

Supplementary Table 1. Table comparing Pearson and Spearman correlation metrics for reduced training sets containing sequences with up to 5 mutations predicting sequences with 7 mutations. Both Pearson and Spearman correlations show similar, limited reductions in correlation as training set size is reduced.

Predict Mutations	LSTM w/RF Pearson	RF Pearson	LSTM w/RF Spearman	RF Spearman
3	0.9	0.93	0.80	0.78
4	0.85	0.9	0.60	0.81
5	0.77	0.82	0.52	0.82
6	0.7	0.68	0.51	0.74
7	0.63	0.48	0.44	0.7
8	0.61	0.22	0.46	0.64
9	0.67	0.02	0.50	0.63
10	0.6	−0.24	0.45	0.51
11	0.6	−0.3	0.48	0.37

Supplementary Table 2. Table comparing Pearson and Spearman correlation metrics for training set containing sequences with up to 2 mutations predicting sequences with 3 to 11 mutations using the LSTM with Random Forest and the Random Forest models. Both Pearson and Spearman correlations show similar reductions in correlation as predictive distance grows.
